# The freetext matching algorithm: a computer program to extract diagnoses and causes of death from unstructured text in electronic health records

**DOI:** 10.1186/1472-6947-12-88

**Published:** 2012-08-07

**Authors:** Anoop D Shah, Carlos Martinez, Harry Hemingway

**Affiliations:** 1 Clinical Epidemiology Group, Department of Epidemiology and Public Health, University College London, London, UK; 2, Consultant Epidemiologist, Frankfurt, Germany

## Abstract

**Background:**

Electronic health records are invaluable for medical research, but much information is stored as free text rather than in a coded form. For example, in the UK General Practice Research Database (GPRD), causes of death and test results are sometimes recorded only in free text. Free text can be difficult to use for research if it requires time-consuming manual review. Our aim was to develop an automated method for extracting coded information from free text in electronic patient records.

**Methods:**

We reviewed the electronic patient records in GPRD of a random sample of 3310 patients who died in 2001, to identify the cause of death. We developed a computer program called the Freetext Matching Algorithm (FMA) to map diagnoses in text to the Read Clinical Terminology. The program uses lookup tables of synonyms and phrase patterns to identify diagnoses, dates and selected test results. We tested it on two random samples of free text from GPRD (1000 texts associated with death in 2001, and 1000 general texts from cases and controls in a coronary artery disease study), comparing the output to the U.S. National Library of Medicine’s MetaMap program and the gold standard of manual review.

**Results:**

Among 3310 patients registered in the GPRD who died in 2001, the cause of death was recorded in coded form in 38.1% of patients, and in the free text alone in 19.4%. On the 1000 texts associated with death, FMA coded 683 of the 735 positive diagnoses, with precision (positive predictive value) 98.4% (95% confidence interval (CI) 97.2, 99.2) and recall (sensitivity) 92.9% (95% CI 90.8, 94.7). On the general sample, FMA detected 346 of the 447 positive diagnoses, with precision 91.5% (95% CI 88.3, 94.1) and recall 77.4% (95% CI 73.2, 81.2), which was similar to MetaMap.

**Conclusions:**

We have developed an algorithm to extract coded information from free text in GP records with good precision. It may facilitate research using free text in electronic patient records, particularly for extracting the cause of death.

## Background

Electronic health records are an important source of information for medical research, but much of the information is stored as unstructured free text rather than in a structured way. Research to date has predominantly used the coded data, which are readily available for analysis, but the free text may contain important additional information relevant to study outcomes, concomitant diseases, procedures, interventions or test results in observational studies [1-4]. Manual review of free text records is time-consuming, so there has been interest in developing software algorithms to extract diagnoses and other clinical information from free text. This is a difficult task, because clinical text can contain a wide range of complex language structures and terminology, and also context-specific abbreviations and acronyms.

Computer programs have been developed to extract specific categories of information from free text including smoking status [5-7], diagnosis of angina or heart failure [3,8], family history [9] and quality of life scores [10]. The MedLEE natural language processing system [11] is used at the Columbia Presbytarian Hospital to encode discharge summaries using ICD-10 codes. The U.S. National Library of Medicine’s MetaMap program [12] is widely used for data mining and indexing of biomedical text. However, overall few programs have been implemented outside the laboratory where they were developed, despite considerable research interest in recent years [13].

The UK General Practice Research Database (GPRD) [14] is a large database of primary care records and is an important source of clinical information for epidemiological and drug safety research. It contains details of consultations, diagnoses, interventions, test results, prescriptions and referrals from general practitioners (GPs) in England, Wales, Scotland and Northern Ireland. GPs code diagnoses using a structured clinical terminology [15]. Currently the ‘Read’ clinical terminology [16] is used, but SNOMED-CT (Systematized Nomenclature of Medicine–Clinical Terms) will be introduced in the next few years [17]. Additional information is entered in free text associated with the coded entries, either typed by the GP or obtained from hospital letters. For prescriptions, the dosage instructions are in the form of free text, and a computer program is used to extract numerical values and dose units for research purposes [18]. However, other types of free text entry in GPRD (such as history and examination findings and hospital discharge summaries) still require manual review if they are to be used in research. An automated system to analyse these texts would greatly facilitate their use in clinical research.

Another potential use of free text in patient records is to investigate the cause of death. For approximately half of GPRD patients in England, the cause of death is available from 2001 onwards by linkage with the national death register at the Office for National Statistics (ONS), but the GP record may be able to contribute information on the circumstances of death (e.g. whether it was a sudden death). A study using the THIN database, another UK general practice database, found that 65% of deaths had the underlying cause clearly recorded in the GP record, but frequently it was in the free text rather than as a Read term [19]. Approximately 150,000 patients in the GPRD died before 2001, and we expect that a large proportion of these patients would have the cause of death recorded in GPRD, but this has not previously been reported in detail.

Our overarching aim was to develop a system for extracting clinical information from free text in electronic patient records. We used cause of death as an initial focus for program development, and have subsequently started to adapt the program to analyse texts associated with other types of clinical event.

## Methods

### Ethics

The GPRD has multi-centre research ethics committee approval for all observational research using GPRD data. Access to the datasets was approved by the GPRD Independent Scientific Advisory Committee (Protocol 09_123R) and the GPRD Scientific Advisory Group (Protocol 648).

### Development of freetext matching algorithm

**Figure 1:**
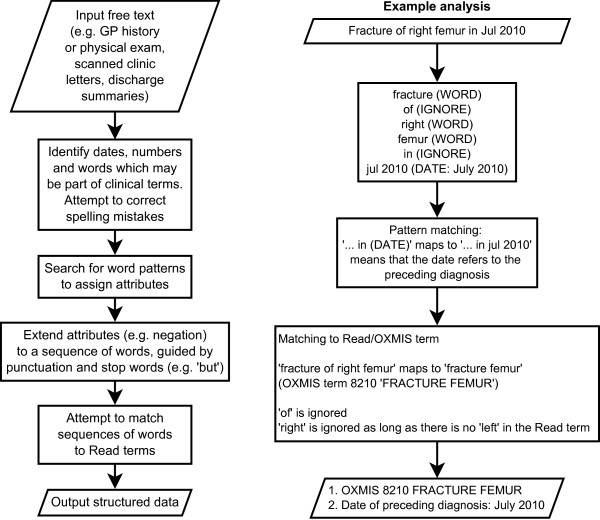
**Flowchart showing how the freetext matching algorithm analyses a text.** Flowchart showing the stages in analysis of a text, with an example of analysis.

We wrote a computer program called the ‘Freetext Matching Algorithm’ (FMA) to extract diagnoses, dates, durations, laboratory results and selected examination findings (heart rate and blood pressure) from unstructured free text. The program uses lookup tables of phrases and synonyms which were created manually. It interprets simple semantic information in the text and Read terms (e.g. negation) to achieve an appropriate match (Figure 1). Unlike the National Library of Medicine’s MetaMap program [12] and MedLEE [20] we chose not to use the Unified Medical Language System [21] in order to minimise the size of our program.

We manually reviewed coded and free text data associated with a random sample of 3310 deaths in the GPRD in 2001, and wrote the initial version of the computer program to extract causes of death from these data. We then extended the functionality to other types of free text. In each development cycle we analysed a random sample of several hundred free text entries from the GPRD, reviewed the output, and modified the program or lookup tables to avoid the errors found on the most recent run.

**Figure 2:**
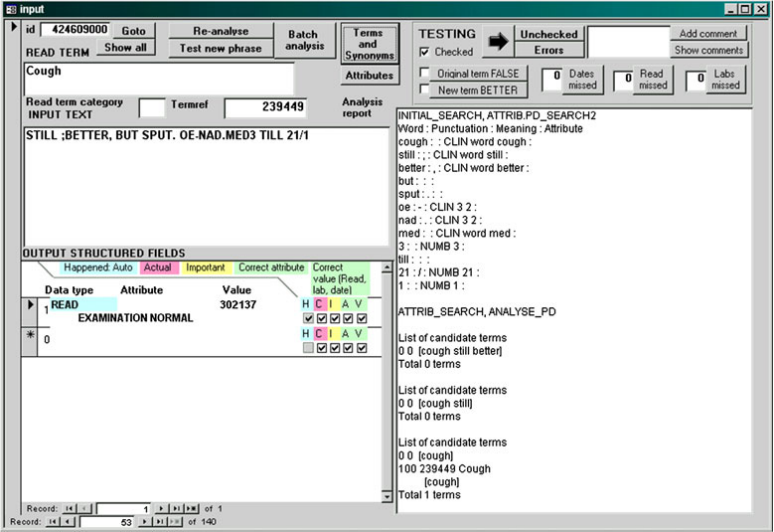
**Dialog box for reviewing results of freetext matching algorithm.** Example of a dialog box in the Microsoft Access 2000 database, showing the interface for analysing a text and reviewing the results.

We implemented the prototype program in Visual Basic 6.0 and developed a user interface in Microsoft Access 2000 in order to facilitate the reviewing process (Figure 2).

### Clinical terminology

The algorithm was principally designed to encode diagnoses in the free text to terms in the Read Clinical Terminology [16], which is used by all primary care practices in the UK. Read terms were designed for coding by GPs and incorporate synonymous terms with variations in the way doctors may express common diagnoses. Apart from diagnoses, the Read terminology includes codes for other categories of information such as history, examination findings, procedures and test results.

FMA includes OXMIS (OXford Medical Information System) terms as well as Read terms. OXMIS was an earlier terminology system used in general practice computer systems from 1987, when GPRD started. Practices switched over to the Read dictionary at varying dates in the 1990s [22]. OXMIS terms have been mapped to Read terms but they may not represent the text exactly, so FMA can output an OXMIS term if it represents the text better than any available Read term. For example, the closest match to the OXMIS term ‘CYSTOCELE’ among Read terms in GPRD is ‘Cystocele without uterine prolapse’ (K510000). However, it is easy to convert OXMIS terms in the output to Read terms if required.

We used a semi-automated process to standardise the wording of Read terms by replacing abbreviations such as ‘a/n’ (antenatal) with the full word, and removing phrases such as ‘NEC’ (not elsewhere classified) which would not be found in ordinary clinical text. We also used a table of patterns to categorise each word in a Read term as positive, negative, optional or ignorable; this would define which words in a Read term need to be present in the text in order for the term to be matched. For example, in order to match the Read term B723z00 ‘Benign neoplasm of bronchus or lung NOS’, a phrase would only need to include one of the words ‘bronchus’ or ‘lung’. We defined a subset of 42,931 Read or OXMIS terms out of the 104,802 in GPRD (as of December 2003) which the algorithm could match to the free text. The majority of terms used by our algorithm (38,981) encoded medical diagnoses; we also included selected terms for symptoms and examination findings, but we excluded terms for administration, procedures or detailed descriptions of causes of injury (e.g. T503.00 ‘Aircraft crash while landing’). We also excluded terms with more than 5 non-ignorable words, as they were too long and complex to match and were infrequently used. Further details of Read and OXMIS term processing are in Additional file 1, pages 10–11.

Our system was designed to enable the easy addition of new codes, which may be useful for coding emerging diseases even before they are recognised in official coding terminologies. To demonstrate this concept, we created the terms ‘Recently in hospital’ and ‘Rhabdomyolysis’ because they were not included in Read or OXMIS, but can encode clinically useful information which may be present in free text.

### Context attributes

It is important to be able to detect if the text states that a diagnosis is negative, but it is difficult to detect negation reliably because of the variety of ways it can be expressed in English. We devised a set of rules to detect negation and other contexts which may apply to words in the text (e.g. whether a diagnosis is past medical history or whether it refers to a family member). These rules are implemented in a table of phrase patterns (‘attrib2’ table), and attributes can be extended to nearby words by a further set of rules written in Visual Basic. For example, the word ‘no’ triggers a ‘negative’ attribute which continues for the length of a list (e.g. ‘no nausea, vomiting or diarrhoea’), and is terminated by ‘but’ or the end of the sentence. Quantitative results are also identified by attributes; for example the text ‘Hb 14.3’ is converted to the value ‘14.3’ with the attribute ‘haemoglobin’. Rules are also used to identify words which might represent names (e.g. ‘Mr. XXX’) and these are given an ‘anonymised’ attribute to force the program to ignore them.

The output of FMA consists of a list of value-attribute pairs (Table 1). There are different sets of valid attributes for Read terms, dates and laboratory results, and only a single attribute can apply to each term or value. Examples of attributes for dates include admission date, date of last menstrual period, or date of preceding diagnosis (see Additional file 1, page 32). Read/OXMIS terms with a blank attribute and no associated date are assumed to refer to a current or past diagnosis for the patient. Other output data types are invalid without an attribute.

**Table 1:** Examples of free text associated with read terms analysed by the freetext matching algorithm

**Analysis mode**	**Read term**	**Free text**	**Structured output**
Standard	55...11 *Angiography - CVS*	coronary severe triple vessel disease + impaired function	Read G340.11 *Triple vessel disease of the heart*
Standard	G20..00 *Essential hypertension*	176/100 but only taking doxaz 4mg od not bd as pw intended - increase to 4mg bd, pn recheck 4w please. otherwise isq but recent diagnosis ca prostate - seeing urol 1w - reassured re treatablility of this nowadays	Systolic BP: 176, Diastolic BP: 100, Follow up: 4 weeks, Read B46..00 *Malignant neoplasm of prostate*
Standard	32...00 *Electrocardiography*	sinus bradycardia (58 bpm) nonspecific intraventricular conduction delay	Read R059.00 *[D]Sinus bradycardia*, Pulse: 58
Standard	55...12 *Angiogram*	ct did not show renal artery stenosis.	Negative: Read P769000 *Renal artery stenosis*
Standard, append	G581.00 *Left ventricular failure*	acute give lasix and o2 and admitted	Read G581000 *Acute left ventricular failure*, Read 8H3Z.00 *Other hospital admission NOS*
Standard, append	14A4.00 *H/O: myocardial infarct* >60	had an mi in 1996	Past medical history: Read G30..00 *Acute myocardial infarction*, date: year 1996
Death	22J..12 *Death*	found dead by family this afternoon, called ambulance, cd 1415hrs, sudden death, rpt to coroner	Read 213100 *[D]Found Dead*, Time: 14:15, OXMIS T4002 *SUDDEN DEATH*
Labtest	42J..00 *Neutrophil count*	original result: ’neutrophil count’ = 4.17 x109 / l (1. 5 - 7. 5)	Lab result: 4.17
Sicknote	9D2..00 *MED5 - doctor’s special stat.*	### 09/01/2007 1 month heart problems, ###	Sickness certificate date: 9-Jan-2007, duration: 1 month
**Examples of errors in interpretation of free text**			
Standard	5853.11 *Echocardiogram*	significant mitral regurg	(no output; ‘regurg’ not recognised as an abbreviation for ‘regurgitation’)
Standard	33B9500 *Exercise tolerance test abnormal*	pt exercised according to the bruce protocol for 4 mins 39secs. he developed chest discomfort during the test with very significant, >3mm, st depression in leads v4, 5 and 6 and leads 2, 3 avf. ###	Read R065600 *[D]Chest discomfort*, Read E2B..00 *Depressive disorder NEC* (incorrect interpretation of ECG finding of ST segment depression)

### How the algorithm works

The text is first ‘cleaned’ by removing computer-generated semi-structured phrases, then converted to lower case and split into individual words (Figure 1). The program identifies dates, numbers and words, and looks up words in dictionaries of ‘medical’ and ‘non-medical’ words. The ‘medical’ words list consists of all words contained in terms in the Read dictionary. The ‘non-medical’ words were taken from the ‘2of4brif’ list of British English words [23] which is in the public domain. If a word does not match any entry in the dictionaries, it is assumed to be mis-spelt, and the program tries to correct the spelling and match it to a dictionary word by inserting or substituting a letter. Any remaining words (which may represent names) are ignored.

The program assigns attributes as described above, then attempts to match sequences of up to five words to Read terms. If the text phrase does not match a Read term exactly, parts of the phrase are substituted by alternative words and phrases using the synonym table. Some words which do not convey clinical information (e.g. ‘of’, ‘the’) are ignored, and the order of the words does not matter as long as they have the same true/false status. ‘Right’ and ‘left’ in the text can also be ignored if they are not included in the Read/OXMIS term; for example ‘fracture right femur’ can match the OXMIS term ‘FRACTURE FEMUR’ (Figure 1). We wrote a function called ‘readscore’ to grade the closeness of a match between a text phrase and a Read term, with a minimum threshold for a satisfactory match (Additional file 1, page 15). Points are deducted from the readscore for each ignorable or negative word not matched, and for use of synonyms rather than identical words. The algorithm tries a number of possible matches for each text phrase, and chooses the match with the highest readscore.

### Analysis modes

Every free text entry in GPRD (and other datasets of UK primary care records) is associated with a Read term, which may be relevant to interpretation of the text. FMA allows some parts of the lookup table and other matching rules to be applied selectively based on the expected information in the free text (Table 2). For example, if the Read term denotes death it is likely that the free text contains the cause of death, so when operating in ‘death’ mode, the program interprets phrases such as ‘1a’ or ‘1b’ before diagnoses as the cause of death category. Alternatively, in ‘pregnancy’ mode, fractions with the denominator 40 (e.g. ‘22/40’) are interpreted as gestational age.

**Table 2:** Analysis modes

**Analysis mode**	**Read term trigger**	**Specific features**
Death	Read term for death	Extract date of death and death certificate categories. Test results are not extracted.
Pregnancy	Read term for pregnancy or text stating ‘pregnant’	Duration in weeks is interpreted as gestational age
Labtest	Read term for test type	A numerical value or ‘normal’, ‘abnormal’ (depending on the test type) can be interpreted as the test result
Normal	Read term for certain investigations (e.g. chest radiograph)	The words ‘normal’ or ‘abnormal’ can be interpreted as the result
Date	Read term stating date	The text is expected to contain a single date
Sicknote	Read term stating ‘MED3’ (sickness certificate) or similar	Dates are regarded as sick certificate start and end dates
Standard	Read term not in one of the above categories	Standard analysis

### Testing

We tested the FMA on a ‘General test set’ containing randomly chosen previously anonymised free text records from a study on coronary artery disease (500 texts from control patients and 500 texts from cases). We also tested FMA in ‘death’ mode on a random sample of 1000 texts associated with Read terms for death or suicide in 2001. None of these texts had been viewed during program development. We used FMA together with an algorithm for selecting the underlying cause of death (described in detail below) to investigate the feasibility of extracting cause of death information from GPRD using automated methods. For this test we used a random sample of 3310 patients who died in 2001.

The aim of the tests was to quantify the recall (sensitivity) and precision (positive predictive value) of detection of Read terms for diagnoses, other Read terms, dates, durations and test results. The algorithm output was reviewed by the first author (ADS, a practising clinician) and this was considered to be the gold standard. A custom interface in Microsoft Access was used to facilitate this process (Figure 2; program code in Additional file 2; Access program in Additional file 3).

The Read or OXMIS term was appended to the free text for the purpose of testing, because this is the way the information is displayed on GPs’ computers. This means that the original Read/OXMIS term would usually be recognised by the program. This term was ignored in the output. Duplicate Read terms in the output and those with attributes for suspected conditions or family history were ignored. A Read term without a specific attribute was considered to represent a current or past event that happened to the patient. When reviewing the correctness of matches, we judged them against the standard that only definite diagnoses or events that applied to the current patient should be coded.

Dates, durations and test results with an incorrect attribute were counted as false positives, and those which were not detected were counted as false negatives.

### Cause of death recording in the GPRD in 2001

The Medical Certificate of Cause of Death (MCCD) is an internationally standardised document filled in by a medical practitioner or coroner to document the cause of a person’s death. Part I of the MCCD contains three categories (Ia, Ib and Ic) for a sequence of up to 3 medical conditions leading to death (e.g. Ia Aspiration pneumonia, Ib Stroke, Ic Atrial fibrillation). Other medical conditions contributing to death but not directly causing it can be listed in Part II. National mortality statistics are compiled by coding each medical condition on the MCCD using the International Classification of Diseases, 10th Edition (ICD-10) since 2000, and selecting a single underlying cause of death according to the ICD-10 selection rules [24]. If the MCCD has been filled in appropriately, the underlying cause of death is usually the lowest lettered entry in Part I.

**Figure 3:**
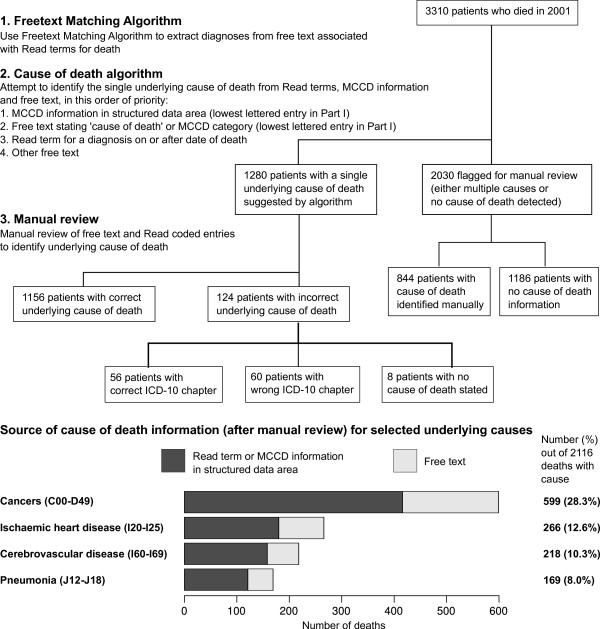
**Source of cause of death information for deaths in 2001.** Flow diagram showing determination of the underlying cause of death for a random sample of 3310 patients who died in 2001

We investigated the recording of cause of death in a random sample of 3310 patients from the GPRD who died in 2001. Cause of death may be recorded by GPs either in a structured data area for recording MCCD diagnoses, or as a Read term on the date of death, or in the free text. We obtained the free text entries associated with Read terms for death, and used FMA to extract diagnoses as Read terms from the free text entries. We mapped all the (original and newly converted) Read terms to ICD-10, then selected the underlying cause using the following algorithm, which we call ‘cause of death algorithm’: If there was only a single cause of death recorded, this was chosen as the cause. If there were multiple entries, diagnoses were extracted from the following sources in decreasing order of priority: MCCD form, followed by free text which explicitly states MCCD categories or ‘cause of death’, then Read terms for diagnoses on or after the date of death, then other free text associated with Read terms for death. If this yielded a single diagnosis, it was chosen as the underlying cause of death. If the MCCD categories were stated (either in the structured area or in the free text), the lowest lettered entry in Part I was selected. In the remaining cases the algorithm flagged that manual review was required (Figure 3).

We reviewed each record manually in order to identify the underlying cause of death (Read terms up to 90 days prior to death, and free text associated with Read terms for death). If there was no cause recorded in the structured data area, Read terms on or after death or the free text, we tried to ascertain the cause of death based on Read terms before the date of death. We accepted such terms only if the cause of death was obvious from the documented sequence of events (e.g. diagnosis of cancer, followed by entries for palliative care, with a final entry stating ‘Death at home’).

### Comparison with MetaMap

Metamap is a natural language processing system which uses the UMLS Metathesaurus to encode free text using a number of possible coding systems [12]. Negation is detected using MetaMap’s implementation of Chapman’s Negex program [25]. We used UMLS Datafile Builder 2011 for Linux [26] to create MetaMap lookup tables for the Read/OXMIS terms used by FMA. We used UMLS MetamorphoSys [27] to prepare an alternative MetaMap vocabulary consisting of the entire ‘Clinical Terms Version 3 (CTV3) (Read Codes), 1999’ which we call ‘Full Read’. As well as diagnoses, this vocabulary includes drugs names, procedures, anatomical terms, and words describing time and location. A complex medical phenomenon may be described by a number of Read terms describing different aspects, e.g. a cancer may be described by separate Read terms for histology, site and grade. Currently GPs in the UK do not code routinely diagnoses with such fine granularity.

We analysed the ‘general’ test set using MetaMap and manually reviewed the results, classifying the extracted terms as correct or incorrect using the same criteria as for assessing FMA. We initially explored combinations of MetaMap analysis options using the ‘Read/OXMIS’ vocabulary and selected the most favourable set of options for testing MetaMap using the ‘Full Read’ vocabulary. We also tested FMA and MetaMap (Full Read) on detection of negation in the Negex test set of 2376 pre-anonymised annotated sentences from clinical reports [28]. All analyses were carried out using the 2011 Linux version of MetaMap; details are given in Additional file 4.

## Results

### ‘General’ test set

FMA correctly extracted 650 positive and 57 negated Read/OXMIS terms from the 1000 free text records. The most common attributes among the positive terms were ‘past medical history’ (5.2%) and ‘cause of death’ (2.2%), but 92.6% of terms were not allocated a specific attribute. For 12 texts (1.2%), the algorithm was able to combine the original Read term and the free text to select a term which was more specific than GP’s original term; for example the term G581.00 ‘Left ventricular failure’ with text ‘acute’ were combined into G581000 ‘Acute left ventricular failure’ (Table 1). For positive diagnoses in the text, precision (positive predictive value) was 91.5% (95% confidence interval (CI) 88.3, 94.1) and recall (sensitivity) was 77.4% (95% CI 73.2, 81.2), giving an F-score of 0.84 (Table 3). Read/OXMIS terms which did not encode diagnoses (such as terms for history, examination and referrals), were also detected with high precision (93.3%; 95% CI 90.0, 95.7).

**Table 3:** Performance of freetext matching algorithm and MetaMap on test sets

			**Algorithm**	**FMA**	**FMA**	**MetaMap**	**MetaMap**
			**Vocabulary**	**Read/OXMIS**	**Read/OXMIS**	**Read/OXMIS**	**Full Read**
			**Test set**	**Death**	**General**	**General**	**General**
			Number of texts	1000	1000	1000	1000
			Number of words	7534	25981	25981	25981
			** Positive diagnoses detected in free text**				
			True positives	683	346	286	273
			False positives	11	32	126	18
			False negatives	52	101	161	174
			Precision, %	98.4 (97.2, 99.2)	91.5 (88.3, 94.1)	69.4 (64.7, 73.8)	93.8 (90.4, 96.3)
			Recall, %	92.9 (90.8, 94.7)	77.4 (73.2, 81.2)	64.0 (59.3, 68.4)	61.1 (56.4, 65.6)
			F-score	0.96	0.84	0.67	0.74
			** Strictly defined precision for positive diagnoses (best term and correct attribute)**				
			Number strictly correct	625	315	260	247
			Precision strict, %	90.1 (87.6, 92.2)	83.3 (79.2, 86.9)	63.1 (58.2, 67.8)	84.9 (80.2, 88.8)
			** Precision of non-diagnosis positive concepts**				
			True positives	84	304	295	453
			False positives	2	22	55	41
			Precision, %	97.7 (91.9, 99.7)	93.3 (90.0, 95.7)	84.3 (80.0, 87.9)	91.7 (88.9, 94.0)
			** Overall precision of positive concepts detected (diagnostic and non-diagnostic)**				
			True positives	767	650	581	726
			False positives	13	54	181	59
			Precision, %	98.3 (97.2, 99.1)	92.3 (90.1, 94.2)	76.2 (73.1, 79.2)	92.5 (90.4, 94.2)
			** Precision of negative concepts detected**				
			True positives	5	57	0	92
			False positives	5	18	0	33
			Precision, %	50.0 (18.7, 81.3)	76.0 (64.7, 85.1)		73.6 (65.0, 81.1)
			** Texts for which algorithm suggested a better Read term than the original term**				
			Percentage of texts	0	1.2	0.5	0.6
			** Dates and durations**				
			True positives	116	96		
			False positives	15	10		
			False negative	25	22		
			Precision, %	88.5 (81.8, 93.4)	90.6 (83.3, 95.4)		
			Recall, %	82.3 (74.9, 88.2)	81.4 (73.1, 87.9)		
			F-score	0.85	0.86		
			** Test results and quantitative measurements**				
			True positives		105		
			False positives		11		
			False negatives		18		
			Precision, %		90.5 (83.7, 95.2)		
			Recall, %		85.4 (77.9, 91.1)		
			F-score		0.89		

Comparison of precision (positive predictive value) and recall (sensitivity) of the Freetext Matching Algorithm (FMA) and MetaMap against the gold standard of manual review, for two test sets: ‘General’, a random sample of 500 texts from cases and 500 from controls in a study on coronary artery disease; and ‘Death’, a random sample of 1000 texts associated with Read terms for death or suicide in 2001.

The texts contained 118 dates and durations, of which the program correctly identified 96. 50% denoted a follow-up interval or date, and 19% specified the timing of event coded by the previous or following Read term. Precision was 88.5% (95% CI 81.8, 93.4), recall was 82.3% (95% CI 74.9, 88.2), and the F-score was 0.85.

The program correctly extracted 105 of the 123 test results or quantitative measurements in the text, including 8 measurements of pulse rate. Precision was 90.5% (95% CI 83.7, 95.2), recall was 85.4% (95% CI 77.9, 91.1) and the F-score was 0.89 (Table 3).

### ‘Death’ test set

FMA performed better on the ‘Death’ test set than on the ‘general’ test set, identifying 683 of the 735 diagnoses giving precision 98.4% (95% CI 97.2, 99.2), recall 92.9% (95% CI 90.8, 94.7), and F-score 0.96 (Table 3). The most common individual Read or OXMIS terms extracted were bronchopneumonia (9.9% of diagnoses), acute myocardial infarction (4.8%) and carcinomatosis (4.0%); see Table 4. Of the terms extracted, 74.4% were not associated with a context attribute, 15.0% specified a MCCD category, and 5.2% stated ‘cause of death’ without a category. FMA also extracted information relating to the circumstances of death. 33 texts (4.2%) stated that the death was sudden (e.g. ‘cardiac arrest’, ‘found dead’, ‘sudden death’ or ‘collapsed’), 19 stated that the patient was hospitalised, and 6 stated that the patient died at home. The program correctly identified 116 of the 141 dates and durations in the test set. Precision was 92.1% (95% CI 85.5, 95.9), recall was 82.3% (95% CI 74.7, 88.0), and F-score 0.87 (Table 3).

**Table 4:** Most common terms extracted by the freetext matching algorithm from texts associated with death in 2001

**Dictionary**	**Term**	**Percentage**
OXMIS	BRONCHOPNEUMONIA	9.9
Read	Acute myocardial infarction	4.8
Read	[*M*]Carcinomatosis	4.0
Read	Ischaemic heart disease	3.4
Read	CVA unspecified	3.1
OXMIS	PNEUMONIA	2.5
OXMIS	UNKNOWN CAUSE	2.5
Read	Congestive cardiac failure	1.7
Read	Septicaemia	1.7
OXMIS	CORONARY ARTERY ATHEROMA	1.5
Read	Lung cancer	1.5
Read	[*X*] Unspecified dementia	1.5
OXMIS	CARCINOMA	1.3
Read	Chronic obstructive pulmonary disease	1.3
OXMIS	HYPERTENSION	1.3
Read	Cardiac arrest	1.1
Read	Deep vein thrombosis	1.1
Read	Left ventricular failure	1.1
Read	Sepsis	1.1
Read	Cerebrovascular disease	1.0

### Cause of death in 2001

The FMA and cause of death algorithm allocated an underlying cause of death for 1280 patients out of 3310 (Figure 3). After manual review of the Read terms and free text for each patient, 2116 (63.9%) had a cause recorded, and 642 patients (19.4%) had the cause of death recorded only in the free text (Table 5).

**Table 5:** Recording of cause of death the GPRD in a random sample of patients who died in 2001

**Source of cause of death**	**Number of patients**	**Percentage**
**information**		
MCCD information in	88	2.7
structured data area		
Read term on or after	1179	35.6
date of death		
Read term before date	207	6.5
of death		
MCCD information in	82	2.5
free text		
Free text stating	43	1.3
cause of death		
Other free text	517	15.6
No cause of death	1194	36.1
information		
Total	3310	100

There were 124 patients for whom the cause of death algorithm suggested an incorrect cause (compared to the gold standard of manual review of the GPRD record). The recall (sensitivity) of FMA and the cause of death algorithm in identifying the correct underlying cause of death was 54.6% (95% CI 52.5, 56.8) and precision was 90.3% (95% CI 88.6, 91.9). However, for 56 of the ‘errors’ the cause suggested by the algorithm was in the correct ICD-10 chapter (e.g. ‘ischaemic heart disease’ instead of ‘myocardial infarction’); thus the precision for choosing the correct ICD-10 chapter was 94.7% (95% CI 93.3, 95.9).

### Comparison with MetaMap

The performance of MetaMap depended on the source vocabulary used; with the same Read/OXMIS terms as FMA and no additional synonyms, it had precision of only 69.4% and 64.0% for positive diagnoses, but using the Full Read vocabulary precision was comparable to FMA (93.8%) and recall was 61.1%. Recall was low because some short and common abbreviations (e.g. ‘MI’ for myocardial infarction) were not recognised by MetaMap. Using the Negex test set, both FMA and MetaMap extracted and assigned a negation status for terms from 1035 test sentences, of which both algorithms were correct in 979 cases, both were wrong in 12 cases, FMA was wrong and MetaMap right in 28 cases and MetaMap was wrong and FMA right in 16 cases. A matched analysis with McNemar’s test on the discordant pairs gave a P value of 0.096, indicating that the difference is not statistically significant. Further details are in Additional file 4.

## Discussion

### Summary of our project

Our novel computer program, the ‘Freetext Matching Algorithm’, can encode 93% of diagnoses in free text associated with Read terms for death in GPRD, and has over 90% precision on a variety of types of free text in GPRD. It may facilitate research using databases of English electronic health records by reducing the need for time-consuming manual review of free text. In the task of extracting diagnoses from clinical text in patient records, the performance of FMA was similar to MetaMap, but MetaMap is more widely applicable to extracting information from other types of biomedical text.

It is often important to use the free text in database studies in order to avoid missing cases, such as death due to a particular cause [19]. The cause of death is available in GPRD for a subset of approximately 50 percent of GP practices in England by linkage to death registrations from 2001 onwards; however for practices not linked to cause of death data in GPRD, previous years, or if a patient dies abroad, researchers have to use the cause as recorded in GPRD. Almost 20% of deaths in the GPRD in 2001 had the cause of death recorded in the free text and not as a Read term (Table 5); if we assume that the proportion is similar for previous years, we estimate that 30,000 patients have the cause of death recorded only in the free text. The FMA can also extract useful information on the circumstances surrounding death, such as timing and suddenness, which are not available in death registration data.

### Limitations

Although our algorithm has the strengths of simplicity and flexibility, it does have important limitations, which we will seek to address with further development. Our algorithm sometimes assigns codes for clinical events which did not happen, or fails to encode clinical events which actually happened to the patient and are stated in the text. The errors occur because free text entries in the GPRD frequently contain spelling errors, abbreviations or complex statements about medical conditions which may or may not apply to the current patient. Our algorithm is conservative and will tend to avoid allocating a code if there is ambiguity in the text, so recall is worse than precision. Performance was better for texts associated with death than with a general sample, probably because texts associated with death frequently contained diagnoses with little extraneous information.

Another limitation is that our algorithm was developed and tested only using free text entries from the GPRD. Further development and testing will be required to ensure that it works on free text from other types of electronic health record (e.g. hospital notes). Our program is written only for UK English; the lookup tables and some aspects of the program would have to be re-written for other languages.

A limitation of our test methodology is that the same person (ADS) wrote the software and reviewed the results of analysis. Also, we reported precision and recall aggregated across the whole test set, rather than separately for specific attributes or diagnoses. It is likely that the accuracy of the algorithm varies by diagnosis, and we have found that it varies based on the source of the free text.

The current implementation of our program is inefficient in its use of computer processing power because it is written in an interpreted language (Microsoft Visual Basic 6.0). On a single-core desktop computer (2.66GHz Intel Celeron, 512MB RAM), it can analyse 2500 words a minute, which is similar to MedLEE (8 seconds per discharge summary on a Sun Blade server with dual 2.1GHz processors [11]). Although our program is adequate for analysing a few thousand texts for specific studies, we aim to make it faster by converting it to a compiled language such as C/C++, in order to enable it to be used at the point of data entry or to analyse millions of records for research (e.g. to find cases of a disease among population records).

### Research implications

The algorithm may be of use for studies investigating specific diseases in GPRD or similar databases of electronic patient records in the UK. Recall for the diagnosis of interest can be improved by adding additional entries to the synonym table. It will reduce the need for costly anonymisation of the free text, but a random sample of texts used in the study would need to be checked manually to verify the accuracy of conversion. An extension of this use is for the program to be run on the entire set of free text in the GPRD, generating a table of newly Read-coded events. This will make it more efficient to select relevant texts for research studies, compared to the current method of searching on text strings.

Apart from diagnoses, the FMA can also extract test or examination results which are not recorded in the coded data. For example, only 10% of GPRD patients have a record of pulse rate in the structured data, but pulse rate was available in 0.8% of texts in our test set (which contained fragments of the electronic record for a few hundred patients). Most of these were quoted in outpatient clinic letters, and it is likely that a large proportion of patients with heart disease have a pulse rate measurement in the free text at some point in their electronic record. It would be almost impossible to extract this information on a large scale without an automated tool such as FMA.

### Clinical implications

With further development, our algorithm may be useful clinically to assist the coding of medical diagnoses. For example, instead of navigating a terminology menu system in order to select an appropriate code, a clinician can simply enter the diagnosis as free text, allow the program to suggest a Read term and confirm that it is correct. Current clinical software systems often have cumbersome time-consuming interfaces for entering structured information, and this hinders the production of structured medical narratives [29,30]. By allowing clinicians to enter information in a way which is natural to them, real-time natural language processing may help in coding diseases that otherwise would not be recorded, thereby improving the completeness of the medical record. This may improve patient safety by making the information easier to retrieve.

Although FMA is not accurate enough to replace a human coder completely, it may save time by enabling coders to concentrate on quality control rather than manually searching for every individual code. An assisted coding system may also overcome the slowness of conventional coding systems in adapting to new diseases, such as toxicity due to novel recreational drugs [31]. A software update may be quicker and easier to deploy than publishing new clinical codes and training staff to use them. Natural language processing might also ease the learning curve in switching to a new coding system, such as the planned transition from Read to SNOMED-CT [17]. Rather than having to learn the new terminology hierarchy, clinicians can continue to enter diagnoses in free text and allow the program to map them to the new codes.

### Further development

We aim to rewrite the software in a faster programming language and remove its reliance on proprietary software. We believe that it is most productive to advance this project using an open source collaborative approach, both for development and to minimise the cost of deployment, and we have therefore licensed our current code under the GNU General Public License Version 3 (Additional files 2 and 3).

We may develop our algorithm to extract details from investigation reports (e.g. coronary angiograms) or to encode therapeutic procedures. We may investigate use of parts of the Unified Medical Language System (UMLS) [21] to be able to detect a wider range of synonyms for diagnostic terms. The UMLS is a comprehensive medical thesaurus maintained by the U.S. National Library of Medicine, which is used by MedLEE [11] and other natural language processing projects. However, as it is such a large database it may significantly increase the size of our program, so its incorporation into our system will have to be considered carefully.

Another desirable feature is for the algorithm to report its confidence in terms selected. Currently, terms may be excluded from the output if there are features in the text which make the diagnosis ambiguous, such as acronyms which can be interpreted in alternative ways. It may be useful to vary the confidence threshold by task; for example identification of texts for manual review requires high recall but precision is less important.

## Conclusions

We have developed a program to extract coded information including causes of death and other diagnoses from free text in electronic patient records. It reduces the need for anonymisation of free text and facilitates the use of these data in research. With further development, it is also likely to be useful in clinical applications such as assisted coding.

## Abbreviations

FMA: Freetext Matching Algorithm; GP: General practitioner; GPRD: General Practice Research Database; NHS: National Health Service;ICD-10: International Classification of Diseases, 10th Edition; MCCD: Medical Certificate of Cause of Death; ONS: Office for National Statistics; OXMIS: Oxford Medical Information System; UK: United Kingdom of Great Britain and Northern Ireland; UMLS: Unified Medical Language System.

## Competing interests

None of the authors have any competing interests to declare.

## Authors’ contributions

ADS analysed the GPRD data for cause of death, designed the Freetext Matching Algorithm, and manually annotated the results of analysis. All authors discussed and reviewed the manuscript.

## Pre-publication history

The pre-publication history for this paper can be accessed here:

http://www.biomedcentral.com/1472-6947/12/88/prepub

## Supplementary Material

Additional file 1 **Documentation for Freetext Matching Algorithm.** PDF document containing general description, user guide and technical documentation. Click here for file

Additional file 2 **Source code of Freetext Matching Algorithm.** ZIP archive containing plain text files of Visual Basic modules in the Access database, and .CSV files (comma separated text files) containing the manually edited lookup tables. The table of Read/OXMIS terms is not included, but is contained in the Access database (Additional file 3). Program code is licensed under the GNU General Public License Version 3. Click here for file

Additional file 3 **Freetext Matching Algorithm in an Access Database.** ZIP archive containing Microsoft Access 2000 database and instructions for use. Program code is licensed under the GNU General Public License Version 3. Click here for file

Additional file 4 **Comparison of Freetext Matching Algorithm with MetaMap and Negex.** PDF document containing details of the MetaMap configuration, performance of different MetaMap options and comparisons of FMA and MetaMap output for selected texts. Click here for file

## Author details

^1^Clinical Epidemiology Group, Department of Epidemiology and Public Health, University College London, London, UK. ^2^Consultant Epidemiologist, Frankfurt, Germany.
