# Supplementary material for: The freetext matching algorithm: a computer program to extract diagnoses and causes of death from unstructured text in electronic health records
Source: BMC Med Inform Decis Mak. 2012 Aug 7;12:88. doi: 10.1186/1472-6947-12-88 (PMC3483188; doi:10.1186/1472-6947-12-88)
Supplement: Additional file 4 — Comparison of Freetext Matching Algorithm with MetaMap and Negex. PDF document containing details of the MetaMap configuration, performance of different MetaMap options and comparisons of FMA and MetaMap output for selected texts. [file 1472-6947-12-88-S4.pdf]

Comparison of Freetext Matching Algorithm with MetaMap and Negex

Anoop D Shah[1], Carlos Martinez[2] and Harry Hemingway[1]

1. Clinical Epidemiology Group, Department of Epidemiology and Public Health, University College London, London, UK 2. Consultant Epidemiologist, Frankfurt, Germany

June 2012

Contents

1 Customisation of MetaMap 2

1.1 Metamap using Full Read source vocabulary . . . 2

1.2 MetaMap using custom Read / OXMIS source vocabulary . . . 2

2 Testing MetaMap 3

2.1 MetaMap using Read / OXMIS source vocabulary . . . 3

2.2 MetaMap using Full Read source vocabulary . . . 4

2.3 Detection of negation in the Negex test set . . . 5

3 Results 6

3.1 Detection of diagnoses and other concepts in GPRD 'general' test set . . . 6

3.2 Detection of negation in the Negex test set . . . 6

4 Examples of output from FMA and MetaMap/Negex 6

5 Appendix: R code for extraction of MetaMap output 12

This document describes tests comparing the U.S. National Library of Medicine's MetaMap natural language processing system [1] with the Freetext Matching Algorithm (FMA). MetaMap incorporates Wendy Chapman's Negex algorithm for detection of negation [2] whereas FMA uses a custom algorithm. By default, MetaMap maps free text to terms from a range of medical source vocabularies supplied with the program ('USAbase'), but it can be restricted to a subset or configured to use a custom vocabulary.

In order to compare the output of the two algorithms, we configured MetaMap so that it would map diagnoses in free text to Read terms, similar to the output from FMA. We used two alternative source vocabularies: either the same set of GPRD Read and OXMIS terms as FMA, or the full Read Clinical Terms Version 3.

We tested the detection of diagnoses and other clinical concepts on a set of 1000 previously anonymised GPRD free text records from cases and controls in a study on coronary artery disease (500 from cases, 500 from controls). This was denoted the 'general' test set. The gold standard was manual review by a clinician (ADS). We also tested the detection of negation in the publicly available Negex test set of annotated anonymised clinical reports [3]. The clinician annotation of 'Affirmed' or 'Negated' was considered the gold standard. We did not test MetaMap on the GPRD 'Death' test set because MetaMap could not recognise death certificate categories in formats such as "1a (diagnosis) 1b (diagnosis) ...", which was common in GPRD and which FMA was specifically programmed to interpret. We wrote a script in R 2.14.1 [4] (see Appendix on page 12) to tabulate the results from MetaMap analyses in order to facilitate manual review.

1 Customisation of MetaMap

1.1 Metamap using Full Read source vocabulary

We used the 2011 Linux version of MetaMap with the 2011AA lexicon. We used the Unified Medical Language System (UMLS) MetaMorphoSys program (2011 version) [5] to generate a UMLS subset containing only the Read Clinical Terms Version 3 (UMLS source code RCD99); 347,577 terms representing 186,073 unique clinical concepts. We suppressed terms of type AA (attribute type abbreviation), AB (abbreviation in any source vocabulary), IS (obsolete synthesized term), OA (obsolete abbreviation) and OP (obsolete preferred term), and specified the order of preference for the remaining term types which MetaMap could map to:

Order	***Source***	***Term type code***	***Description***
1	Read	PT	preferred term
2	Read	SY	designated synonym
3	Read	AT	attribute type
4	Read	AS	attribute type synonym

We ran MetaMap Datafile Builder (2011 Linux version) [6] to produce the lookup tables with word variants for the MetaMap 'strict' data model.

1.2 MetaMap using custom Read / OXMIS source vocabulary

We generated source tables for Datafile Builder using the 42,933 terms used by FMA (32,186 Read, 10,745 OXMIS and 2 manually created terms). We considered that each term represented a distinct

clinical concept. We generated the tables MRCON (term text, one row per term), MRSO (containing the Read/OXMIS code, one row per term), MRSTY (semantic type of each term), MRRANK (order of preference of sources, stating Read preferred to OXMIS) and MRSAB (source abreviations). We did not consider any of the terms to be suppressible. We used the same synonym file ('SM.DB') as for the Full Read configuration. We used MetaMap Datafile Builder (2011 Linux version) [6] to generate MetaMap lookup tables under the 'strict' model.

2 Testing MetaMap

2.1 MetaMap using Read / OXMIS source vocabulary

We tested MetaMap using our custom list of Read and OXMIS terms under all possible combinations of the following options [7]:

--composite_phrases 3 causes MetaMap to construct longer, composite phrases from the simple phrases produced by the parser. This allows phrases such as "pain *on the* left side *of the* chest" to map to a single concept 'Left sided chest pain' rather than separate concepts as it would without the option. The integer option is the maximum number of prepositional phrases that will be added to a noun, and we used the value of 3 as advised in the MetaMap 2011 Release Notes [8]

--ignore_word_order allows MetaMap to ignore the order of words in the phrases it processes

--word_sense_disambiguation causes MetaMap to attempt to disambiguate among concepts scoring equally well in matching input text (e.g. whether the word "cold" refers to a low temperature or coryzal illness)

--allow_concept_gaps allows MetaMap to retrieve candidates with gaps (such as 'Unspecified childhood psychosis' for 'unspecified psychosis')

--unique_acros_abbrs_only allows the generation of acronym/abbreviation variants if they have unique expansions

The 32 sets of output Read/OXMIS terms from these combinations were merged into a single results table for comparison with output from FMA. Any discrepancies were checked manually by a clinician (ADS). As with the FMA tests described in the main article, the aim was to assess recall and precision in the detection of positive diagnoses. Other Read terms matched (e.g. symptoms, referrals) were assessed for precision only.

Manual review was against the standard that a term without any other context information should represent a current or past diagnosis for that patient. A term was considered 'strictly correct' if it was the best availble term for the concept. A term was considered 'correct' but not 'strictly correct' if it was correct but not the best available map (e.g. "breast cancer" mapped to the OXMIS term 'CANCER'). If the correct interpretation of a term is negative, or it does not apply to the current patient (e.g. the term refers to a family member or advice), it was considered to be a false positive.

The combination of options which produced best performance was used in testing MetaMap with the full Read dictionary.

2.2 MetaMap using Full Read source vocabulary

As well as diagnoses, the full Read dictionary contains terms for temporality, laterality, body parts etc. which can match fragments of text in isolation but may not convey clinically useful information (for example, the Read term 'Disease' could match any mention of the word "disease" in the text). Therefore we restricted the output to Read terms with the following semantic types and other Read terms extracted from the same phrase (which might give additional contextual information):

- Acquired Abnormality
- Acquired Abnormality, Disease or Syndrome
- Anatomical Abnormality
- Congenital Abnormality
- Disease or Syndrome
- Environmental Effect of Humans, Hazardous or Poisonous Substance
- Finding
- Injury or Poisoning
- Laboratory or Test Result
- Mental or Behavioral Dysfunction
- Mental Process
- Neoplastic Process
- Organ or Tissue Function
- Pathologic Function
- Phenomenon or Process
- Sign or Symptom

For example, if the text 'chest pain' was analysed as a single phrase and mapped to the concepts 'Chest [body part]' and 'Pain [Sign or Symptom]', they were assessed as jointly conveying the meaning of the text. We ignored any output Read terms which did not fit these criteria, and also ignored terms consisting of numbers / fractions or those which were ambiguous or did not convey useful information: 'Afraid', 'Awareness', 'Carries', 'Confidence', 'Drive', 'Feelings', 'Finding', 'Forgetful', 'Gifted', 'Happy', 'Hopelessness', 'Nothing', 'Opposition', 'Palpation', 'Planning', 'Recognition', 'Related', 'Runs', 'Sad'. For example, the term 'Forgetful' may be extracted from texts such as "he forgot to pick up the prescription", which does not necessarily imply that the patient is generally forgetful.

We used the MetaMap analysis options: **`--composite_phrases 3 --word_sense_disambiguation --ignore_word_order --unique_acros_abbrs_only`**. This combination of options was chosen because it had the best performance when tested using the Read/OXMIS source vocabulary, apart from word sense disambiguation which had no effect.

The results were reviewed manually by a clinician (ADS) and classified as 'correct' if there was no error and 'strictly correct' if the term or combination of terms represented the entire meaning of the diagnosis in the text (including context). The precision of detection of positive diagnoses, positive non-diagnosis

terms and negated terms was assessed separately. Recall was assessed for positive diagnoses only, to be consistent with the standard of testing for FMA.

2.3 Detection of negation in the Negex test set

The Negex algorithm classifies word or phrases in the text as negated or affirmed. However, FMA and MetaMap output a list of mapped terms, and negation applies to terms in the output rather than words in the text. Some sentences did not contain any concepts that were mapped to Read or OXMIS terms, so the program could not produce any information about negation for this sentence.

We tested the detection of negation in the publicly available Negex test set of 2376 anonymised sentences from 120 clinical reports of 6 types (emergency department, discharge summaries, surgical pathology, radiology, operative notes, echocardiograms) [3]. This dataset already contains a clinician annotation of 'Negated' or 'Affirmed' for each sentence. The negation status applies to a particular 'condition' (e.g. a diagnosis, symptom or examination finding) in the text, and the rest of the sentence might give clues as to its negation status. The algorithm (FMA or MetaMap) was initially applied to the 'condition' to map it to one or more Read or OXMIS terms. If a map was found, the whole sentence was interpreted and the negation states of terms which were also extracted from the 'condition' text were processed as follows:

- All positive, or single positive term – compare with supplied annotation; if 'Affirmed' the result is correct, if 'Negated' it is incorrect
- All negative, or single negative term – compare with supplied annotation; if 'Negated' the result is correct, if 'Affirmed' it is incorrect
- Some positive, some negative – manual review by a clinician (ADS), as some of the terms may not be relevant. For example, using the text "IMPRESSION: NO EVIDENCE OF ACUTE CARDIOPULMONARY DISEASE.", MetaMap extracted 'Acute (Temporal Concept) TRUE' and 'Diseases (Disease or Syndrome) FALSE', of which 'Diseases' was considered to be the relevant concept.
- No match – manual review by a clinician, as the terms detected from the whole sentence may be different to those from the text of the condition in isolation

MetaMap using our custom Read/OXMIS source vocabulary was unable to detect negation, so MetaMap was tested using the 'Full Read' and 'USAbase' source vocabularies. The MetaMap analysis options were: **`--composite_phrases 3 --word_sense_disambiguation --ignore_word_order --unique_acros_abbrs_only`**, as per the tests on GPRD free text. The FMA attributes 'negative', 'negpmh' (negative past medical history) and 'negfamily' (negative family history) were considered negative, and all other attributes or a blank attribute were considered positive.

The results for FMA and MetaMap were presented both separately (for all terms detected by the algorithm), and as a matched analysis restricted to terms mapped by both algorithms. For the matched analysis we tested the null hypothesis that if the algorithms gave different results, the correct algorithm was equally likely to be FMA as MetaMap (McNemar's test). Hypothesis tests and confidence intervals for proportions were calculated using the exact binomial distribution in R 2.14.1 [4].

3 Results

3.1 Detection of diagnoses and other concepts in GPRD 'general' test set

Metamap using custom Read / OXMIS source vocabulary

Allowing composite phrases and ignoring word order improved recall when MetaMap with the custom Read/OXMIS vocabulary was tested on the GPRD 'general' test set (Table 1). Allowing concept gaps resulted in worse precision. Allowing unique abbreviations and acronyms resulted in two fewer incorrect non-diagnosis terms when analysed with composite phrases and allowing concept gaps, but made no difference otherwise. The results were identical whether or not the word sense disambiguation option was used.

The set of options with the highest F-score was to allow composite phrases, ignore word order and not allow concept gaps. This combination achieved 64% recall and 69% precision on positive diagnoses (see Table 3 in the main article).

MetaMap using Full Read source vocabulary

Performance was better using the 'Full Read' source vocabulary, with precision 94% and recall 61% on detection of positive diagnoses, and precision 92% for positive non-diagnosis terms (see Table 3 in the main article). Negated terms were detected with precision 74%.

3.2 Detection of negation in the Negex test set

Out of the terms extracted by each algorithm, both FMA and MetaMap correctly detected the negation status of the term in about 95% of cases, but MetaMap extracted more terms than FMA (Table 2).

Both algorithms extracted the relevant terms from about half of the test sentences. In the subset for which the algorithms gave different results, FMA was correct in 36% of cases versus MetaMap Full Read (P=0.10), and FMA was correct in 56% of cases versus MetaMap with USAbase (P=0.43). Overall there was no statistically significant difference in the probability of either algorithm being correct among terms extracted by both algorithms.

4 Examples of output from FMA and MetaMap/Negex

Example 1

Text (from GPRD free text, 'general' test set): "Magnetic resonance imaging demonstrated bone metastases involving his spine but with no impending cord compression."

FMA output (using standard options):

Read/OXMIS term	*Attribute*	*Readscore*
OXMIS 1989M: METASTASIS		98
Read F29y400: Cord compression	negative	100

Table 1: Performance of MetaMap in coding diagnoses and other concepts in 1000 ‘general’ free text records from GPRD, using the custom Read/OXMIS vocabulary with various analysis options.

Analysis options				***Diagnosis terms***							***Non-diagnosis terms***			
Composite phrases	Ignore word order	Allow abbrev.	Allow gaps	Number strictly correct	Number correct (true pos)	Number incorrect (false pos)	Number missed (false neg)	***Recall***	***Precision***	***F-score***	Number correct (true pos)	Number incorrect (false pos)	***Precision***	***Overall Precision***
			●	232	271	195	176	60.6%	58.2%	0.594	275	84	76.6%	66.2%
		●	●	232	271	195	176	60.6%	58.2%	0.594	275	84	76.6%	66.2%
	●		●	261	288	187	159	64.4%	60.6%	0.625	298	83	78.2%	68.5%
	●	●	●	261	288	187	159	64.4%	60.6%	0.625	298	83	78.2%	68.5%
●			●	262	291	194	156	65.1%	60.0%	0.624	309	96	76.3%	67.4%
●		●	●	262	291	194	156	65.1%	60.0%	0.624	309	94	76.7%	67.6%
●	●		●	269	296	200	151	66.2%	59.7%	0.628	317	100	76.0%	67.1%
●	●	●	●	269	296	200	151	66.2%	59.7%	0.628	317	98	76.4%	67.3%
				224	262	135	185	58.6%	66.0%	0.621	268	52	83.8%	73.9%
		●		224	262	135	185	58.6%	66.0%	0.621	268	52	83.8%	73.9%
	●			250	276	130	171	61.7%	68.0%	0.647	281	54	83.9%	75.2%
	●	●		250	276	130	171	61.7%	68.0%	0.647	281	54	83.9%	75.2%
●				250	279	125	168	62.4%	69.1%	0.656	290	54	84.3%	76.1%
●		●		250	279	125	168	62.4%	69.1%	0.656	290	54	84.3%	76.1%
●	●			260	286	126	161	64.0%	69.4%	0.666	295	55	84.3%	76.2%
●	●	●		260	286	126	161	64.0%	69.4%	0.666	295	55	84.3%	76.2%

Table 2: Negation status of terms extracted from the Negex test set as detected by FMA and two configurations of MetaMap

Gold standard	***MetaMap Full Read*** Affirmed	Negated	None	***MetaMap USAbase*** Affirmed	Negated	None	***FMA*** Affirmed	Negated	None
Affirmed	1716	27	142	1725	24	136	853	14	1018
Negated	88	319	84	84	349	58	28	206	257
% correct	94.7 (95% CI 93.6, 95.6)			95.1 (95% CI 94.1, 95.9)			96.2 (95% CI 94.9, 97.2)		

Table 3: Number of terms with correct and incorrect detection of negation status by FMA and MetaMap, for terms in the Negex test set detected by both algorithms

FMA	***MetaMap Full Read*** Correct	Incorrect	***MetaMap USAbase*** Correct	Incorrect
Correct	979	16	939	32
Incorrect	28	12	25	11
P value (McNemar's test)	0.096		0.427	

FMA codes 'bone metastases' to a more general term for metastases, and recognises that the 'Cord compression' concept is negated.

MetaMap output (using the 'Full Read' vocabulary; candidates not shown):

```
metamap11.BINARY.Linux (2011)

Control options:
  lexicon_year=2011
  mm_data_year=2011AA
  composite_phrases=3
  word_sense_disambiguation
  unique_acros_abbrs_only
  negex
  bracketed_output
  mm_data_version=11_read
|: Established connection to Tagger Server on localhost.
Established connection to WSD Server on localhost.

NEGATIONS:

Processing 00000000.tx.1: Magnetic resonance imaging demonstrated bone metastases
involving his spine but with no impending cord compression.

Phrase: "Magnetic resonance imaging demonstrated bone metastases"
>>>>> Phrase
magnetic resonance imaging demonstrated bone metastases
<<<<< Phrase
>>>>> Mappings
Meta Mapping (730):
```

```
   561 Magnet [Manufactured Object]
   632 Resonance [Qualitative Concept]
   774 Bony metastasis (Metastasis to bone) [Neoplastic Process]
<<<<< Mappings

Phrase: "involving"
>>>>> Phrase
involving
<<<<< Phrase
>>>>> Mappings
Meta Mapping (916):
   916 Involvements [Functional Concept]
<<<<< Mappings

Phrase: "his spine"
>>>>> Phrase
spine
<<<<< Phrase
>>>>> Mappings
Meta Mapping (1000):
  1000 Spine [Body Part, Organ, or Organ Component]
<<<<< Mappings

Phrase: "but with no impending cord compression."
>>>>> Phrase
but with no impending cord compression
<<<<< Phrase
>>>>> Mappings
Meta Mapping (790):
   790 Cord compression (Umbilical cord compression) [Pathologic Function]
<<<<< Mappings
```

In this example MetaMap suggests a more specific term than FMA for bone metastases, but Negex does not classify the ‘Cord compression’ concept as negated.

Example 2

Text (from Negex test set): “GU - No frequency, urgency or dysuria”

FMA output (using standard options):

Read/OXMIS term	***Attribute***	***Readscore***
Read J11y.00: Unspecified gastric ulcer		92
Read 1A25.00: Urgency	negative	100
OXMIS 7860C: DYSURIA	negative	100

The word “GU” is incorrectly interpreted as an abbreviation for ‘gastric ulcer’, but the readscore is low because the synonym entry has a low ‘priority’ denoting ambiguity. Urinary frequency is not detected because ‘urinary’ is not stated explicitly in the text.

MetaMap output (using the 'Full Read' vocabulary; candidates not shown):

```
metamap11.BINARY.Linux (2011)

Control options:
  lexicon_year=2011
  mm_data_year=2011AA
  composite_phrases=3
  word_sense_disambiguation
  negex
  bracketed_output
  mm_data_version=11_read
|: Established connection to Tagger Server on localhost.

NEGATIONS:
Negation Type:     nega
Negation Trigger:  no
Negation PosInfo:  5/2
Negated  Concept:  C0013428:Dysuria
Concept  PosInfo:  30/7


Processing 00000000.tx.1: GU - No frequency, urgency or dysuria

Phrase: "GU -"
>>>>> Phrase
gu
<<<<< Phrase

Phrase: "No frequency,"
>>>>> Phrase
frequency
<<<<< Phrase
>>>>> Mappings
Meta Mapping (1000):
  1000 Frequency [Quantitative Concept]
<<<<< Mappings

Phrase: "urgency"
>>>>> Phrase
urgency
<<<<< Phrase
>>>>> Mappings
Meta Mapping (928):
   928 Urgent [Temporal Concept]
<<<<< Mappings

Phrase: "or"
```

```
>>>>> Phrase
<<<<< Phrase

Phrase: "dysuria"
>>>>> Phrase
dysuria
<<<<< Phrase
>>>>> Mappings
Meta Mapping (1000):
  1000 Dysuria [Sign or Symptom]
<<<<< Mappings
```

MetaMap/Negex only detects negation of ‘Dysuria’. Frequency and urgency are mapped to generic terms rather than urinary symptoms. The word ‘GU’ is ignored.

Example 3

Text (from Negex test set): “The patient denied any HEADACHE.”

FMA output (using standard options):

Read/OXMIS term	*Attribute*	*Readscore*
OXMIS 791: HEADACHE		100

```
metamap11.BINARY.Linux (2011)

Control options:
  lexicon_year=2011
  mm_data_year=2011AA
  composite_phrases=3
  word_sense_disambiguation
  negex
  bracketed_output
  mm_data_version=11_read
|: Established connection to Tagger Server on localhost.

NEGATIONS:
Negation Type:     nega
Negation Trigger:  denied
Negation PosInfo:  12/6
Negated  Concept:  C0018681:Headache
Concept  PosInfo:  23/8


Processing 00000000.tx.1: The patient denied any HEADACHE.

Phrase: "The patient"
>>>>> Phrase
patient
```

```
<<<<< Phrase

Phrase: "denied"
>>>>> Phrase
denied
<<<<< Phrase

Phrase: "any HEADACHE."
>>>>> Phrase
headache
<<<<< Phrase
>>>>> Candidates
Meta Candidates (1):
  1000 Headache [Sign or Symptom]
<<<<< Candidates
>>>>> Mappings
Meta Mapping (1000):
  1000 Headache [Sign or Symptom]
<<<<< Mappings
```

FMA recognised ‘denies’ but not ‘denied’. This can be corrected by amending the attrib2 table of (patterns for context detection) to recognise the word ‘denied’ for negation. However Negex correctly detects negation of the headache symptom.

5 Appendix: R code for extraction of MetaMap output

MetaMap was run in a Linux virtual machine (Xubuntu 12.04). In order to analyse text, first the tagging server and the word sense disambiguation server were started using these shell commands (run from the **public_mm** base directory):

```
./bin/skrmedpostctl start
./bin/wsdserverctl start
```

Then the following R functions were used to analyse each text and extract the mapping from the human readable output into an R data.frame.

```
# A utility function to concatenate text
"%&%" <- function(a, b){paste(a, b, sep='')}

# Path to our installation of MetaMap
METAMAP_PATH <- '~/Documents/public_mm/bin/metamap11'

################################################################
# extract_info: A function to extract information from MetaMap
#               human-readable output
#
extract_info <- function(metamap_output){
```

```
  # Argument: metamap_output is a character vector containing
  #           human readable MetaMap output
  # Find which lines of the output list negated concepts
  negations <- metamap_output[grep('^Negated  Concept', metamap_output)]

  # Generate a vector of CUIs of negated concepts
  negconcepts <- unlist(lapply(strsplit(negations, ':'),
    function(x){gsub(' ', '', x[2])}))
  # Locate start and end of mappings output
  mapstart <- which(metamap_output=='>>>>> Mappings')
  mapend   <- which(metamap_output=='<<<<< Mappings')

  # Extract lines of output which contain concept mappings
  include <- rep(0, length(metamap_output))
  for (i in seq_along(mapstart)){
    include[(mapstart[i]+1):(mapend[i]-1)] <- i
  }
  include[grep('^Meta Mapping', metamap_output)] <- 0
  order <- include[include > 0]
  strings <- metamap_output[include > 0]

  # Extract term text
  text <- sub('[ C[:digit:]]+:([[:print:]^\\[]+) \\[[[:print:]]+', '\\1', strings)
  # Extract CUI (Concept Unique ID)
  cui <- sub('[ [:digit:]]+(C[[:digit:]]+)\\:[[:print:]]+', '\\1', strings)
  # Extract semantic type
  sty <- sub('[[:print:]]+\\[([-, [:alnum:]]*)\\]$', '\\1', strings)
  # Whether or not the term is negated
  true <- !(cui %in% negconcepts)

  # Combine the results into a data.frame for output
  data.frame(order=order, text=text, cui=cui, sty=sty, true=true,
    stringsAsFactors=FALSE)
}

################################################################
# metamap: Function to do MetaMap analysis for a specified text
#
metamap <- function(text, dataversion='USAbase',
  analysisoptions=' --composite_phrases 3 --ignore_word_order ' %&%
    '--word_sense_disambiguation --unique_acros_abbrs_only ',
  outputoptions=' --bracketed_output --negex --show_cuis '){
  # Produces an R object with MetaMap output
  out <- system('echo "' %&% text %&%
    '" | ' %&% METAMAP_PATH %&% analysisoptions %&% ' '
    %&% outputoptions %&% ' --mm_data_version ' %&% dataversion,
    intern=TRUE, ignore.stderr=TRUE)
  result <- extract_info(out)
```

```
  result[!duplicated(result),]
}
```

References

[1] Aronson A: **MetaMap**. U.S. National Library of Medicine 2011, [http://metamap.nlm.nih.gov/].

[2] Chapman WW, Bridewell W, Hanbury P, Cooper GF, Buchanan BG: **A Simple Algorithm for Identifying Negated Findings and Diseases in Discharge Summaries**. *Journal of Biomedical Informatics* 2001, **34**(5):301 – 310, [http://www.sciencedirect.com/science/article/pii/S1532046401910299].

[3] Chapman W: **Negex Test Set**. 2009, [http://code.google.com/p/negex/wiki/TestSet].

[4] R Development Core Team: **R: A Language and Environment for Statistical Computing**. R Foundation for Statistical Computing, Vienna, Austria 2011, [http://www.R-project.org/]. [ISBN 3-900051-07-0].

[5] **UMLS®Reference Manual**. U.S. National Library of Medicine, Bethesda (MD) 2009, [http://www.ncbi.nlm.nih.gov/books/NBK9683/].

[6] Rogers W, Lang FM, Gay C: **MetaMap Data File Builder**. U.S. National Library of Medicine 2012, [http://metamap.nlm.nih.gov/].

[7] US National Library of Medicine: **MetaMap 2009 Usage** 2009, [http://metamap.nlm.nih.gov/MM09_Usage.shtml].

[8] US National Library of Medicine: **MetaMap 2011 Release Notes**. 2011, [http://metamap.nlm.nih.gov/].
